# Prediction of Radiotherapy Compliance in Elderly Cancer Patients Using an Internally Validated Decision Tree

**DOI:** 10.3390/cancers14246116

**Published:** 2022-12-12

**Authors:** Biche Osong, Inigo Bermejo, Kyu Chan Lee, Seok Ho Lee, Andre Dekker, Johan van Soest

**Affiliations:** 1 Department of Radiation Oncology (Maastro), GROW School for Oncology, Maastricht University Medical Centre+, 6229 ET Maastricht, The Netherlands; 2 Department of Radiation Oncology, Gil Medical Center, Gachon University College of Medicine, Incheon 21565, Republic of Korea

**Keywords:** elderly cancer patients, radiotherapy, compliance, decision tree

## Abstract

**Simple Summary:**

The developed decision tree uses patient status, the Charlson comorbidity index, the Eastern Cooperative Oncology Group Performance scale, age, sex, cancer type, health insurance status, radiotherapy aim, and fractionation type to distinguish between compliant and noncompliant patients. The developed tree’s ability to identify those patients who are likely to discontinue their radiotherapy treatment is reasonably good, providing caregivers with a rationale for deciding whether to start radiotherapy treatment or look for alternative treatment for these patients. Additionally, the developed decision tree can help to boost treatment compliance by targeting those patients who are likely to discontinue therapy with incentives and techniques to help them adhere to treatment, especially for patients already receiving therapy.

**Abstract:**

This study aims to analyze the relationship between the available variables and treatment compliance in elderly cancer patients treated with radiotherapy and to establish a decision tree model to guide caregivers in their decision-making process. For this purpose, 456 patients over 74 years of age who received radiotherapy between 2005 and 2017 were included in this retrospective analysis. The outcome of interest was radiotherapy compliance, determined by whether patients completed their scheduled radiotherapy treatment (compliance means they completed their treatment and noncompliance means they did not). A bootstrap (B = 400) technique was implemented to select the best tuning parameters to establish the decision tree. The developed decision tree uses patient status, the Charlson comorbidity index, the Eastern Cooperative Oncology Group Performance scale, age, sex, cancer type, health insurance status, radiotherapy aim, and fractionation type (conventional fractionation versus hypofractionation) to distinguish between compliant and noncompliant patients. The decision tree’s mean area under the curve and 95% confidence interval was 0.71 (0.66–0.77). Although external validation is needed to determine the decision tree’s clinical usefulness, its discriminating ability was moderate and it could serve as an aid for caregivers to select the optimal treatment for elderly cancer patients.

## 1. Introduction

Approximately 60% of cancer incidence occurs in adults aged 65 and older [1,2,3,4,5]. Nowadays, many consider people over 70 years as elderly [4]. However, this group of patients, generally called the elderly, are often not granted access to therapeutic clinical trials [6,7]. Therefore, there are still many unanswered questions regarding the clinical and behavioral responses of elderly cancer patients to cancer treatment [2]. Generally, elderly persons are frail due to their relatively weaker immune systems [8]. This frailty, combined with common multiple comorbidities, makes them vulnerable to chronic illnesses and even therapy [9,10], making it difficult to choose the appropriate treatment [2,11,12]. Although studies have shown that some treatments are feasible for elderly patients [13,14], the likelihood of their discontinuation is greater than that of the younger generation [15]. This is primarily because of increased comorbidity, decreased performance status, and overall poor health due to treatment [16].

Radiotherapy, a treatment option that uses ionizing radiation to treat various malignant and benign disorders with curative or palliative intent, is one of the most widely used and effective cancer treatments. However, the treatment process is sometimes not completed as planned, with unwanted interruptions encountered during the treatment process either due to technical reasons or patient-related reasons, such as religious beliefs, financial burden, radiotherapy myths, and travel burden [17,18,19]. Such treatment interruptions may affect local control and overall survival [20] and induce unnecessary treatment-related toxicities for these patients [20,21,22].

Several studies have looked at treatment compliance as an outcome of interest either for a particular disease or a combination of diseases [15,16,23]. However, as far as we are aware, these studies have focused their analysis on a univariate association of the available variables with the endpoint treatment compliance in (elderly) cancer patients. Therefore, a strategy or model to identify elderly cancer patients who might not complete their planned radiotherapy treatment beforehand would be beneficial.

Decision trees, a commonly used prediction technique, are more suitable to model such endpoints as they can connect several variables to naturally classify patients into various risk groups based on the outcome of interest and present the knowledge graphically to serve as a decision aid [24].

Therefore, this study aimed to use the available patient information to develop a decision tree that can discriminate between elderly cancer patients based on their planned radiotherapy treatment completion and use the visual knowledge as a decision support system for physicians and caregivers [25,26].

## 2. Patients and Methods

After obtaining ethical approval from the Internal Review Board, data for 456 cancer patients above 74 years of age (elderly) who were treated with radiotherapy were retrospectively collected from the patient’s medical records at the Gil Medical Center in Korea between January 2005 and September 2017. All tasks were performed following relevant guidelines and regulations.

Radiotherapy was performed using 6 to 15 MV X-ray photons or electron beams with various energies. Compliance, the outcome of interest, was defined as the completion of the prescribed radiotherapy dose, and noncompliance was defined as the discontinuation of therapy by the patient without the caregivers’ advice or consent. Only patients with lung, metastatic, head and neck, and gastrointestinal and hepatobiliary cancer were enrolled in this study because they are prevalent cancer types and have a high rate of noncompliance with treatment.

The total radiotherapy dose, fractionation, and radiotherapy field size were determined based on the type of cancer. Patient information such as age, sex, Eastern Cooperative Oncology Group Performance scale (ECOG PS) [27], Charlson comorbidity index (CCI) [28], patient status (in-patient or out-patient), radiotherapy aim, fractionation type, health insurance status, and cancer type were considered in this study. The radiotherapy aim and fractionation type (conventional fractionation and hypofractionation) were considered for treatment information. Only patients treated with conventional fractionation and hypofractionation techniques were included in this study. The stereotactic radiosurgery (SRS) / stereotactic body radiotherapy (SBRT) technique was not included because only one patient was noncompliant with radiotherapy (Supplementary Materials, Figure S1). Based on radiotherapy dose and fractionation, patients were classified into either the conventional fractionation (1.8–2.0 Gy fractionation) group, hypofractionation (more than 2 Gy fractionation) group or stereotactic radiosurgery/ stereotactic body radiotherapy (more than 7.5 Gy fractionation) group.

Health insurance status was used as a surrogate for the actual financial status of the patient. In Korea, the health protection system exempts economically disadvantaged people (patients with medical care insurance) from the insurance premiums which means they do not have to pay for their treatment. Medical insurance status was introduced to indirectly estimate the economic status of elderly patients, which was classified into two patient groups, one with health insurance and the other with medical care in Korea. Patients with health insurance pay part of the treatment fee, and the government pays the rest, whereas, for medical care patients, the government pays the entire treatment cost for the patients.

## 3. Decision Trees

A decision tree is a nonlinear discrimination method that uses independent variables to split a sample into progressively smaller subgroups, by utilizing binary rules. The basic idea is to recursively partition the covariate space to form subgroups called nodes on the tree for subjects with similar characteristics based on the outcome of interest. The iterative procedure begins with the independent variable with the strongest association and with the dependent variable of interest based on specific criteria [24]. The first variable or node at the top of the tree is called the root node, which is the tree’s most important feature. The other variables that help the tree split further are the internal nodes. Each variable split on the tree is called a branch or edge, and the end of the branch that does not split any further is called the decision or leaf node; in this case, whether a patient completed (compliance) or discontinued (noncompliance) their planned radiotherapy treatment [24,29].

## 4. Statistical Analysis

Exploratory analyses and data visualization such as distribution and box plots were applied to gain insights into the data sets and understand the data’s underlying patterns. The bootstrap (B = 400) technique and grid search option within the caret package [30] was employed to find the best tuning parameters. The identified optimal hyperparameters were then used to grow the decision tree to predict compliance in elderly cancer patients. The performance of the resulting decision tree was evaluated using a five-fold cross-validation method. The respective area under the curve (AUC) and calibration for each of the folds were then computed and plotted. The odds ratios at each branch of the tree were computed to measure the association between treatment compliance and the variable. All statistical analyses were performed with the R software, and a *p* value < 0.05 was considered statistically significant.

## 5. Results

A total of 456 elderly cancer patients with a median age of 78 (74–92) years who received radiation therapy were considered in order to grow and internally validate the decision tree. Table 1 shows the demographic and clinical characteristics of the participants in the study. There was no difference in the median age or CCI for compliant and noncompliant patients. In contrast, the median fractionation and radiotherapy dose for compliant patients was three-fold that of noncompliant patients. Metastatic patients were the highest contributors to this study based on sample size, and lung cancer patients were the highest contributors in terms of noncompliance.

Focusing only on noncompliant patients (Table 1), as it is the group of interest, 87 (19.1%) patients out of the 456 analyzed did not complete their planned radiotherapy treatment (Supplementary Materials, Figure S2). More than 50% of the patients in this population did not complete their treatment due to their worsening performance status. The rest of the patients decided not to continue treatment (21%), had radiotherapy-related morbidity (8%), or died during treatment (2%).

Based on Table 1, men had a higher noncompliance rate than women. Only 6 (11.3%) patients with poor (3-4) ECOG PS did not adhere to their treatment against 81 (20.1%) with good (0–2) ECOG PS. There were similar observations for health insurance status, as there were 79 (19.8 %) patients with health insurance but only 8 (13.8 %) with medical care. The number of patients treated with curative intent (67 (19.6%)) was three times more than that treated with palliative intent (21(18.1%)). The number of noncompliant inpatients was only slightly higher than that of outpatients while the number of those treated with conventional fractionation 53 (18.0%) was more than those treated with hypofractionation 34 (21.1%). With respect to cancer type, lung and metastatic cancer had the highest number of noncompliant patients with 25 (19.7%) and 25 (17.6%) respectively while head and neck cancer had the least with 14 (23.3%).

**Figure 1:**
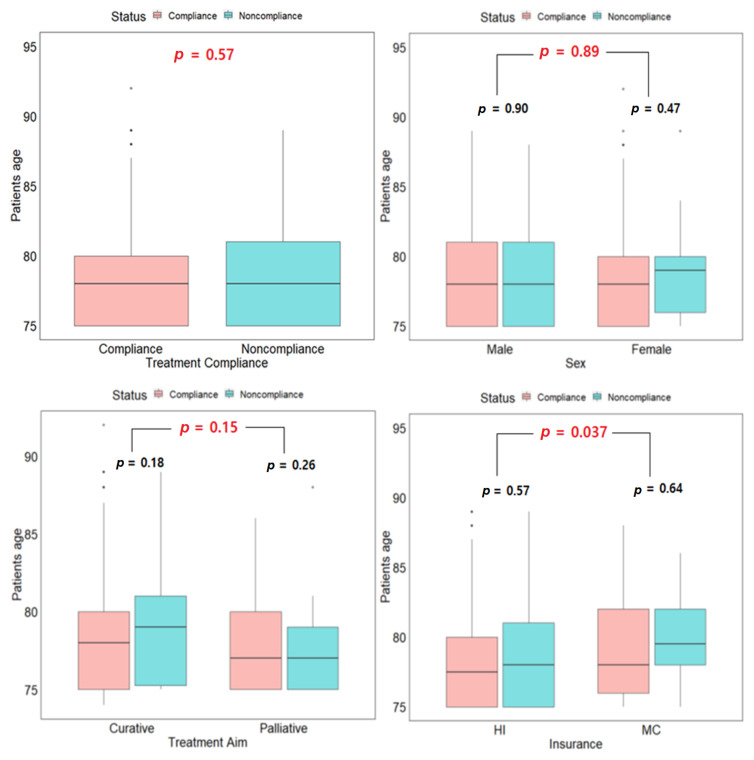

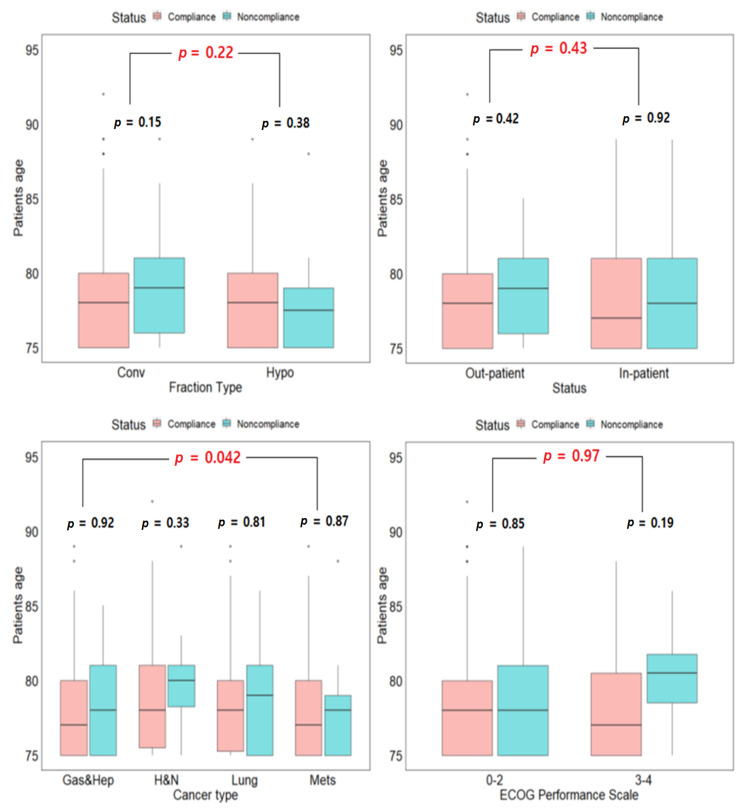
Box-plot of age and all the considered variables by compliance status. *p*-values in black are for the within-group comparison and red represents the between-group comparison. HI: Health insurance, MC: Medical care, Conv: Conventional fractionation, Hypo: Hypofractionation, Gas&Hep: Gastrointestinal and Hepatobiliary, H&N: Head and Neck, Mets: Metastatic, ECOG: Eastern Cooperative Oncology Group.

Given that this study was centered around elderly cancer patients, box plots of age and the other considered variables were produced to visualize the age distribution within these variables by compliance status. Figure 1 shows that there was no significant mean age difference between compliant and noncompliant patients. The same nonsignificant result was observed for all the other variables except for health insurance status and tumor type. Additionally, there was no significant mean age difference within the variables’ levels.

### Decision Tree

**Figure 2:**
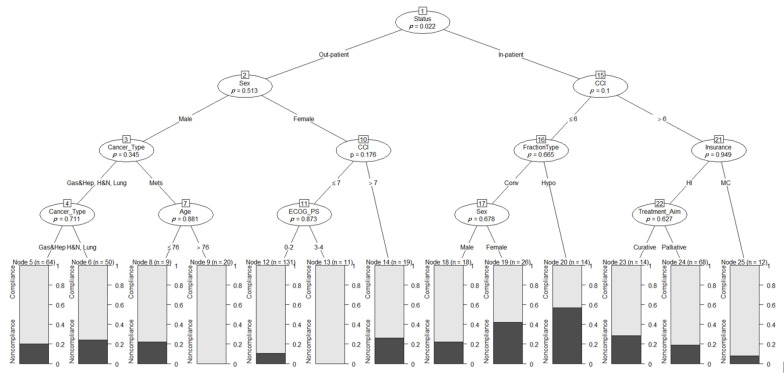
Decision tree for predicting radiotherapy compliance in elderly cancer patients. The oval structures represent the variables that branch out to form the tree. The branches connect the variables and hold the condition for the splits. The rectangle structures that do not branch any further on the tree are the leaf or decision node from which the probability of compliance (white) and noncompliance (black) is read. The values at the top of the leaf nodes indicate the number of patients in that node, and the p-values in the oval structures indicate the significance level of the variable.

Based on the bootstrap result (Supplementary Materials, Figure S4), a maximum tree depth of 4 was selected with a minimum criterion of 0.041. Figure 2 shows the resulting decision tree with these optimal tuning parameters from the bootstrap runs. The oval structures are the independent variables represented as a condition or node, based on which the tree splits into branches or edges. The black text between the thin line on both sides of the nodes describes the split situation that is to be followed to obtain the patient’s probability of radiotherapy compliance from the rectangular structures at the bottom of the tree.

To use the decision tree as a decision tool, locate the root node on the top of the tree and read the condition. Then, follow a series of repeated IF-THEN processes based on the patient characteristics on the decision tree until you arrive at the last node, which splits no further. The patients’ probability of compliance or noncompliance is then read from the leaf node. On this tree, the most important variable is the patient’s status, which splits between inpatients and outpatients. For outpatients, we take the left route, which indicates that the sex of the patient is needed to reach a decision.

In contrast, if the patient is an inpatient, we take the right path, where the second most essential variable for this group of patients needs to be consulted. Here, we checked the patient’s CCI. If the patient has a CCI value above 6, we move right, where the patient’s insurance status becomes important and split into patients with health insurance and medical care. For medical care patients, their corresponding compliance probability is read directly from the leaf node at the bottom of the tree. In this case, there is an approximately 95% chance that the patient will complete their planned radiotherapy treatment or a 5% chance of noncompliance. On the other hand, if the patient has health insurance, we move left where the treatment aim becomes important, and the tree splits directly into two leaf nodes, with curative patients having a slightly higher probability of noncompliance (~25%) compared to palliative patients (20%).

**Figure 3:**
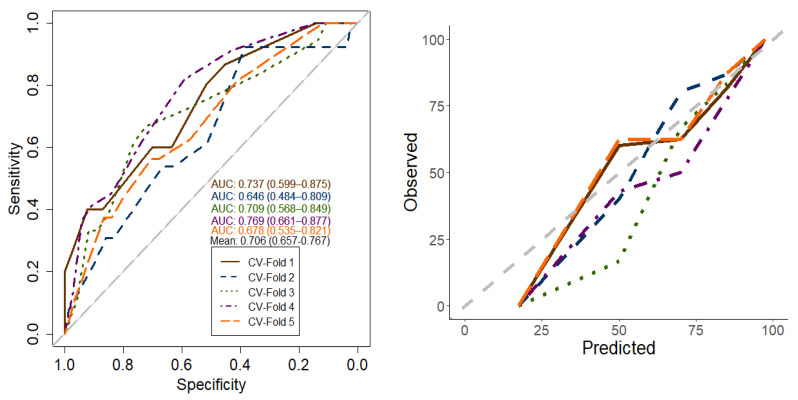
Decision tree performance in terms of discrimination (AUC) and calibration.

Figure 3 shows the decision tree’s performance for predicting radiotherapy compliance in elderly cancer patients based on the area under the curve (AUC) and the calibration plot. The decision tree’s mean AUC and 95% confidence interval (CI) were 0.71 (0.66–0.77). The sensitivity and specificity of the developed decision tree were 0.64 and 0.75, respectively, based on a threshold of 0.19. These figures imply that approximately 64% of the patients who completed their planned radiotherapy treatment were correctly classified by the tree as patients who adhered to treatment. On the other hand, 75% of the patients who discontinued their treatment were correctly identified by the tree as noncompliant.

Calibration plots indicate how similar the predicted probabilities are to the actual or observed values. For a perfect or ideal model, all the points should fall precisely on the dotted diagonal gray line. The plot showed good agreement between the observed and predicted probabilities for most of the cross-validation samples.

## 6. Discussion

The decision tree methodology was the preferred analysis method because it simplifies the complex relationships between the dependent and target variables and makes the connections easier to understand and interpret [26]. Unlike other classification models, tree models are more intuitive, self-explanatory, and easy to understand. Decision trees are nonparametric, meaning assumptions or data distributions do not tie them down [24,33]. Therefore, they can be applied to any data to evaluate and account for complex relationships within the data and present the results in a (clinically) usable form [17,33]. Their ability to naturally classify patients into various groups based on the outcome or endpoint of interest makes them a very appealing and handy decision tool in medicine [33].

The current study developed and internally validated a decision tree for predicting radiotherapy compliance in elderly cancer patients. The decision tree had a mean AUC value of 0.71 (0.66–0.77), with patient status, sex, cancer type, age, CCI, ECOG PS, fractionation type, treatment aim, and insurance as essential factors for determining radiotherapy compliance. Our findings are similar to Gupta et al. [16], who also analyzed treatment compliance in cancer patients and reported that age, sex, tumor stage, concurrent chemoradiotherapy, and travel distance were significantly associated with noncompliance with radiotherapy. However, it is somewhat challenging to make direct comparisons and interpretations of both studies because of the difference in the analytical approaches and study design.

In our study, the CCI was a key factor for predicting radiotherapy compliance in elderly patients for both inpatients and outpatients. However, for outpatients, it was only necessary to determine radiotherapy compliance for females. Other studies have also found the CCI to be a significant factor affecting adherence to treatments in females, such as chemotherapy and radiotherapy in breast cancer [34]. Nonetheless, Di Genesio Pagliuca et al. [13] found no statistically significant correlation between the CCI and chemotherapy in a mixed population of 137 (60% males and 40% females) elderly patients treated with chemoradiotherapy. These contradictory results on the importance of the CCI to predict treatment compliance can be attributed to the difference in the patient population under consideration. The decision tree shows that CCI is a pivotal factor in treatment compliance, but for certain patient groups. Therefore, without proper subgroup analysis or methods such as decision trees, important predictor variables could be easily missed, leading to contradictory results. In addition, the study did report that most of the patients who stopped chemoradiotherapy had a relatively higher CCI and poor performance status. In this study, the odds of noncompliance for an outpatient female with a CCI below 7 was only 0.25 times the odds of an outpatient female with a CCI value above 7, while the noncompliance odds for inpatients with a CCI value below 6 was 2.55 times the odds of noncompliance for inpatients with a CCI value above 6 (Supplementary Materials, Table S1) and all the odds ratios were statistically significant.

This study could not include information about the morbidity of elderly cancer patients because of too much missing information. Hence, we could not evaluate the relationship between morbidities and radiotherapy compliance. Nevertheless, we postulate that good and poor ECOG PS patients could not complete their scheduled radiotherapy due to treatment-related morbidities. However, patients with a poor ECOG PS would be more affected by radiotherapy treatment than those with a good ECOG PS. In the present study, patients with a poor ECOG PS had a lower median age in the compliance group than in the noncompliance group (Figure 1). This age difference was absent for patients with a good ECOG PS. Based on the developed decision tree, the ECOG PS is only pivotal for outpatient females with a CCI value of less than 7. Patients with a good ECOG PS have a relatively higher noncompliance probability than those with poor ECOG PS. In general, except for the ECOG PS before radiotherapy, numerous factors can affect a patient’s ECOG PS during treatment. These include oral mucositis and esophagitis, both of which are typical side effects of radiotherapy either for head and neck cancer or lung cancer. In a study by Yoon et al. [35], the authors reported that the main reasons for the discontinuation of radiotherapy treatment in elderly lung cancer patients were that five patients (42%) experienced aggravation of their general condition and cancer progression, and seven patients (58%) experienced treatment-related toxicity. They concluded that physicians should pay attention to selecting elderly cancer patients and chemotherapy agents considering general conditions and toxicity before planning concurrent chemoradiotherapy.

In terms of indirectly estimating the economic status of elderly patients, we analyzed radiotherapy compliance according to medical insurance status, classified as patients with health insurance and patients with medical care. As per our institution’s policy, insured patients who decide to receive radiotherapy should pay the treatment fee at every visit and not the total cost at the initial appointment. Generally, radiotherapy treatment fees are higher than the other treatment modalities, which means that cancer patients need for radiotherapy funds is still substantial relative to the other treatments and can cause significant “financial distress.” Therefore, patients with health insurance are more likely to have a financial burden than patients with medical care since the government covers the treatment costs. As a result, the noncompliance rate in the health insurance group was tenfold higher than that in patients with medical care. Although there was a statistically significant age difference between these two insurance groups, noncompliant patients had a higher median age within each group, especially for medical care patients (Figure 1). Based on the decision tree, inpatients with a CCI value greater than six and medical care have a higher compliance probability than patients with health insurance. Also, at this node (node 21), the odds of noncompliance for an inpatient with a CCI above 6 is reduced by approximately 60% if the patient has medical care insurance (Supplementary Materials, Table S1) For health insurance patients, compliance is mostly affected by the type of treatment they receive, with patients treated with curative intent having a relatively higher noncompliance probability than palliative patients (Figure 2). More so the odds of noncompliance for patients who received palliative care (node 22) treatment was 0.58 times the odds of noncompliance for curative treatment patients (Supplementary Materials, Table S1).

The developed decision tree results can help caregivers and physicians in their decision-making process. For instance, an 80-year-old female not admitted to the hospital (outpatient) and suffering from a metastatic disease is expected to complete his planned radiotherapy schedule based on the decision tree. In such a case, the physician can recommend the most appropriate radiotherapy technique for the patient regardless of age by considering the feasibility of completing treatment based on objective results from the decision tree. On the other hand, an outpatient female with a CCI value above 7 has a 25% chance of not completing her planned radiotherapy treatment. Such information can assist in selecting the most appropriate treatment for this patient or channeling the right resources for a better outcome. Generally, when radiotherapy treatment lasts longer than seven weeks, it induces fatigue in patients. This is especially true for the elderly, particularly as their physical and mental status is impacted. Hence, based on the decision tree, physicians could propose a shorter radiotherapy treatment period technique, such as stereotactic body radiotherapy (SBRT), for patients predicted to be noncompliant with radiotherapy treatment.

In a study by Amini et al. [36], the authors used SBRT with a relatively short duration of treatment compared to conventional split radiotherapy for elderly head and neck cancer patients with poor performance status. Hayashi et al. [37] also reported the effects of SBRT in elderly patients with stage I non-small-cell lung cancer (NSCLC). They concluded that SBRT for stage NSCLC was well tolerated and feasible in very elderly patients, although elderly patients experienced significantly more severe radiation pneumonitis. Recent advances in radiotherapy techniques that reduce radiation-related adverse effects, such as intensity-modulated radiation therapy, SBRT, and stereotactic radiosurgery, should be considered for treating elderly cancer patients. Although we did not enroll SRS/SBRT-treated patients in this study, we did visualize the distribution of compliant and noncompliant patients within the different fractionation types, which shows that all but one patient was compliant with radiotherapy treatment as expected (Supplementary Figure S1).

In this study, the number of outpatients who discontinued their planned radiotherapy treatment was higher than inpatients, with a significant difference in the odds of noncompliance (OR = 0.48, 95% CI [0.29–0.78]) between the two patient groups (Supplementary Materials, Table S1). This outpatient noncompliance majority was more pronounced in the lung and gastrointestinal/hepatobiliary cancer patients (Supplementary Materials, Figure S3). Typically, extended fractionation schedules will require outpatients to commute between the radiotherapy center and their residence. Since elderly patients are more susceptible to treatment-related fatigue and deficits in physical activity, the constant travel can severely impact their quality of life, making it infeasible for them to come for future treatments. To increase adherence to planned radiotherapy treatment in this patient group, Palwe et al. [25] suggested providing accommodation for these patients closer to the treatment center or having more frequent outpatient visits after the third week of planned radiotherapy treatment. Contrary to these two cancer types, the number of noncompliant outpatients with metastatic or head and neck cancer was lower or equal to inpatients respectively (Supplementary Materials, Figure S3). A possible explanation for this difference could be that the general condition of patients in this group is relatively poor and can deteriorate easily or the radiation treatment was administered mainly to relieve severe metastasis-related symptoms such as pain and neurologic symptoms. This increases the chance of a patient discontinuing treatment if symptoms were immediately alleviated during treatment or could be advised by a caregiver if the patient’s condition worsens.

To the best of our knowledge, this study is the first to assess the predictive value of decision trees for radiotherapy compliance in elderly cancer patients. Therefore, there is room for improvement. As a retrospective study derived from a single institution, this analysis could not include other information, such as morbidity, during radiotherapy. External validation of the developed decision tree is therefore needed to ascertain its clinical usefulness. Nonetheless, the performance of the developed decision tree to determine if a patient will complete their planned radiotherapy treatment is better than tossing a coin. In addition, 75% of the patients who discontinued their treatment were correctly identified by the tree as noncompliant. This, enables caregivers to closely monitor a subgroup of patients to help prevent them from discontinuing their treatment, which might lead to an unnecessary increase in treatment cost and time wasted for both the patient and caregiver.

## 7. Conclusions

In conclusion, we have developed and internally validated a decision tree to predict radiotherapy compliance in elderly cancer patients. Based on the decision tree, treatment compliance mainly depends on the patient’s status. Other clinical, social, demographic, and treatment features, such as CCI, ECOG PS, age, sex, cancer type, health insurance status, radiotherapy aim, and fractionation type, also influenced treatment compliance. The developed tree’s ability to identify those patients who are likely to discontinue their radiotherapy treatment is reasonably good, providing caregivers with a better rationale for deciding whether to start radiotherapy treatment or look for alternative treatment for these patients. The developed decision tree has moderate discriminating ability and it could serve as an aid for caregivers to select the optimal treatment for elderly cancer patients; however external validation is needed to determine its clinical usefulness. Additionally, the developed decision tree can also help boost treatment compliance by targeting those patients who are likely to discontinue therapy with incentives and techniques to help them adhere to treatment, especially for patients already receiving therapy.

## Figures and Tables

**Table 1:** General patient demographic and clinical characteristics.

Variable	Levels	Compliance	Noncompliance	Total
Age (year)	Median (^1^ sd)	78 (3.5)	78 (3.3)	78 (3.5)
Fractionation	Median (sd)	25 (9.9)	8 (7.2)	25 (11)
Radiotherapy dose (Gy)	Median (sd)	50.4 (15.4)	16.5 (13.5)	45 (18.9)
(^2^ EQD2)	Median (sd)	50 (15.0)	17.7 (13.5)	44.25 (18.7)
^3^ CCI	Median (sd)	6 (1.9)	6 (1.9)	6 (1.9)
Sex	Male	187 (78.9%)	50 (21.1%)	238 (52.0%)
	Female	182 (83.1%)	37 (16.9%)	219 (48.0%)
^4^ ECOG PS	0–2	322 (79.9%)	81 (20.1%)	403 (88.4%)
	3–4	47 (88.7%)	6 (11.3%)	53 (11.6%)
Patient Status	Out-patient	258 (84.9 %)	46 (15.1%)	304 (66.7%)
	In-patient	111 (73.0 %)	41 (27.0%)	152 (33.3%)
Radiotherapy aim	Curative	274 (80.6 %)	66 (19.4%)	340 (74.6%)
	Palliative	95 (81.9 %)	21 (18.1%)	116 (25.4%)
Health insurance status	Medical care	50 (86.2 %)	8 (13.8 %)	58 (12.7%)
	Health insurance	319 (80.2 %)	79 (19.8 %)	398 (87.3%)
Fraction type	Conventional	242 (82.0%)	53 (18.0%)	295 (64.7%)
	Hypofraction	127 (78.9 %)	34 (21.1%)	161 (35.3%)
Cancer type	Lung	102 (80.3%)	25 (19.7%)	127 (27.9%)
	Metastatic	117 (82.4%)	25 (17.6%)	142 (31.1%)
	Head & Neck	46 (76.7%)	14 (23.3%)	60 (13.1%)
	Gastrointestinal & Hepatobiliary	104 (81.9%)	23 (18.1%)	127 (27.9%)
Total		369 (80.9%)	87 (19.1%)	456 (100%)

^1^ sd: Standard Deviation, ^2^ EQD2: Equivalent Dose in 2 Gy fractions, ^3^ CCI: Charlson comorbidity index, ^4^ ECOG PS: Eastern Cooperative Oncology Group Performance Scale. Biological Effective Dose (BED) [31] and Equivalent Dose in 2 Gy fractions (EQD2) [32] using an α/β ratio of 10 were also used to quantify radiobiological concepts into concrete interpretable values.

## Data Availability

The data presented in this study are available on request from the corresponding author. The data are not publicly available due to legal issues.

## Supplementary Materials

The following supporting information can be downloaded at: https://www.mdpi.com/article/10.3390/cancers14246116/s1, Figure S1: Box and Bar plots for fractionation type by compliance status; Figure S2: Reasons for radiotherapy noncompliance; Figure S3: Bootstrap output for selecting optimal parameters; Figure S4: Bar plots of compliance by patients status for each disease; Table S1: The odds ratio of noncompliance with the corresponding 95% confidence interval and *p*-value for each branch on the developed decision tree to predict compliance in elderly cancer patients.
