# Supplementary material for: Prediction of Radiotherapy Compliance in Elderly Cancer Patients Using an Internally Validated Decision Tree
Source: Cancers (Basel). 2022 Dec 12;14(24):6116. doi: 10.3390/cancers14246116 (PMC9776371; doi:10.3390/cancers14246116)
Supplement: Supplementary file 1 [file cancers-14-06116-s001.zip › Supplementary Table S1.pdf]

The odds ratio of noncompliance with the corresponding 95% confidence interval and p-value for each branch on the developed decision tree to predict compliance in elderly cancer patients.						
**Node**	**Variable**	**Levels**	**Noncompliance**	**Compliance**	**Odds ratio (95% CI)**	**P-value**
**1**	Patient Status	Out-patient	46	258	0.48 (0.29 - 0.78)	<0.05
		In-patient	41	111		
**2**	Sex	Female	19	142	0.57 (0.30 - 1.08)	0.08
		Male	27	116		
**3**	Cancer type	Metastatic	02	27	0.28 (0.04 - 1.04)	0.06
		Others	25	89		
**4**	Cancer type	Lung	11	38	1.05 (0.42 - 2.60)	0.90
		Others	14	51		
**7**	Age	Above 76	00	20	-	-
		Below 76	02	07		
**10**	CCI	Below 7	14	128	0.25 (0.09 – 0.91)	0.03
		Above 7	05	14		
**11**	ECOG-PS	3-4	00	11	-	-
		0-2	14	117		
**15**	CCI	Below 6	23	35	2.55 (1.32 – 5.67)	<0.05
		Above 6	18	76		
**16**	Fraction type	Conv	15	29	0.39 (0.10 - 1.37)	0.12
		Hypo	18	06		
**17**	Sex	Male	04	14	0.40 (0.09 - 1.52)	0.17
		Female	11	15		
**21**	Insurance	MC	01	11	0.39 (0.01 - 2.28)	0.31
		HI	17	65		
**22**	Treatment aim	Palliative	13	55	0.58 (0.16 - 2.49)	0.43
		Curative	04	10		
