# Supplementary figures and images for: Prediction of Radiotherapy Compliance in Elderly Cancer Patients Using an Internally Validated Decision Tree
Source: Cancers (Basel). 2022 Dec 12;14(24):6116. doi: 10.3390/cancers14246116 (PMC9776371; doi:10.3390/cancers14246116)

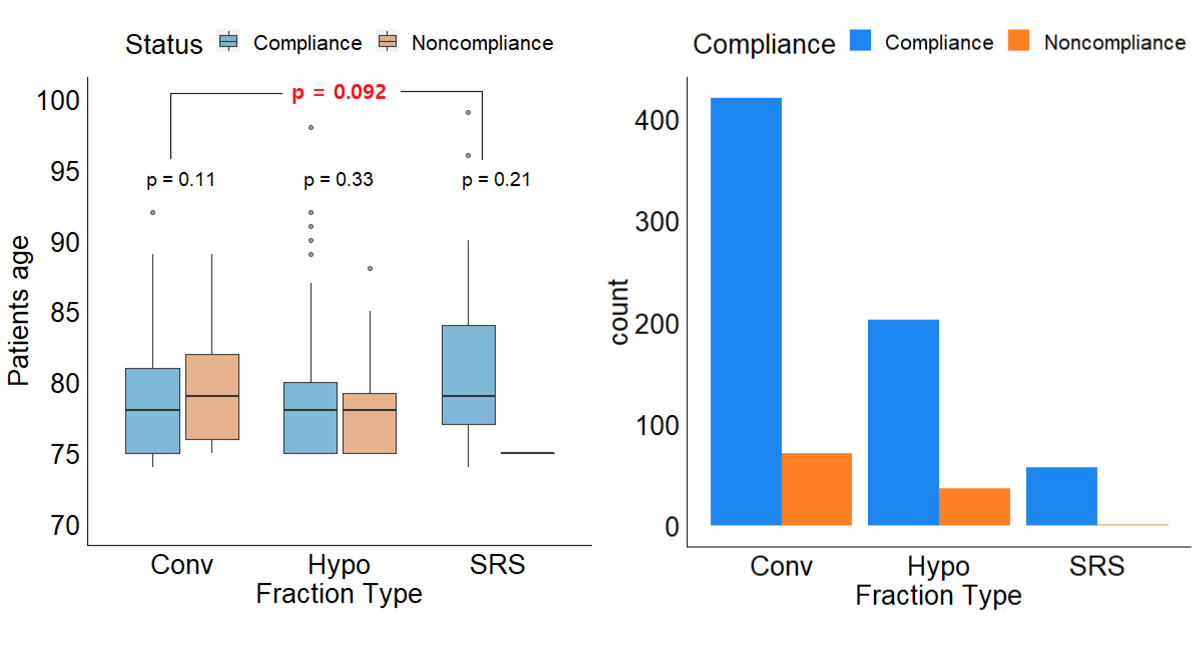

Supplement: Supplementary file 1 [file cancers-14-06116-s001.zip › Supplementary Figure S1.png]

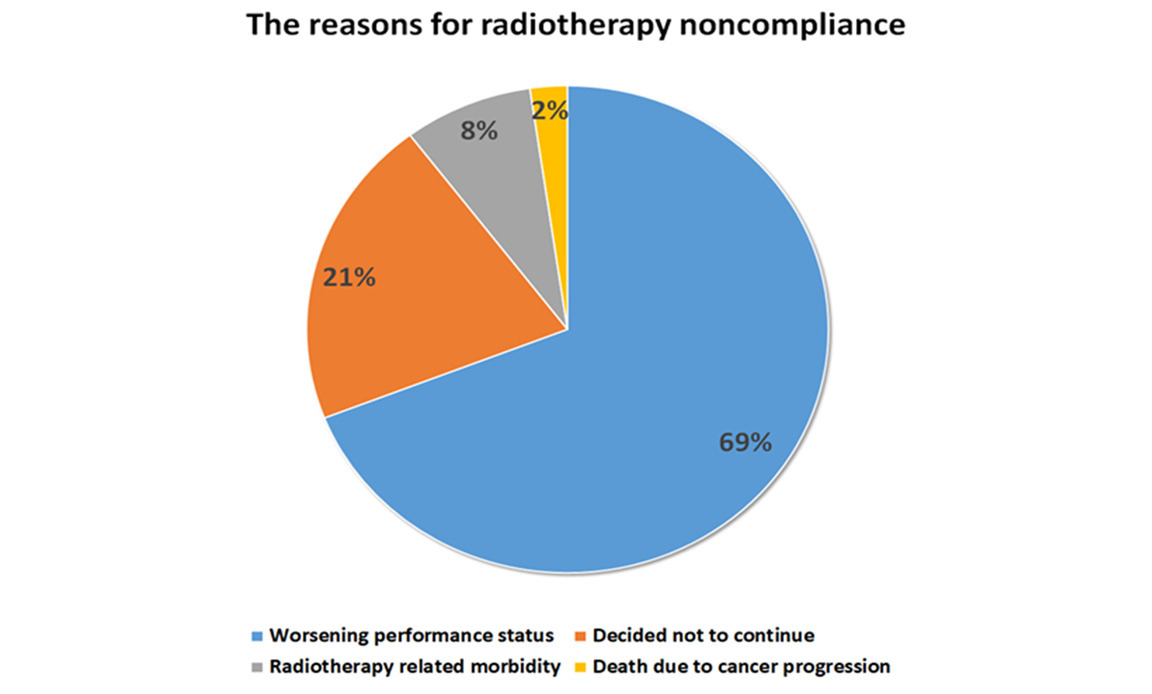

Supplement: Supplementary file 1 [file cancers-14-06116-s001.zip › Supplementary Figure S2.png]

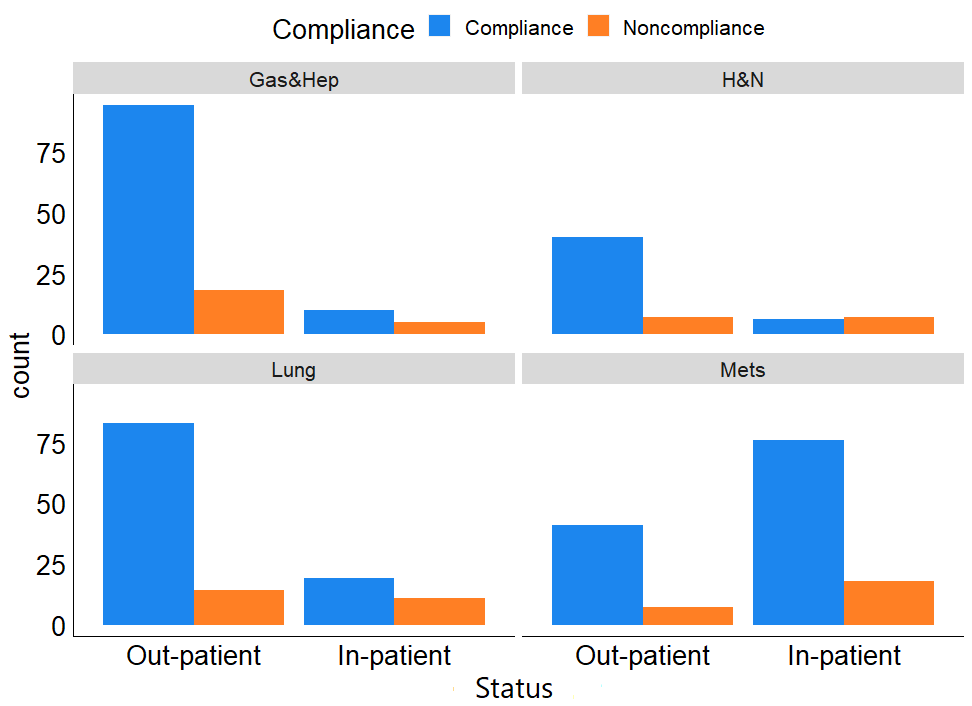

Supplement: Supplementary file 1 [file cancers-14-06116-s001.zip › Supplementary Figure S3.png]

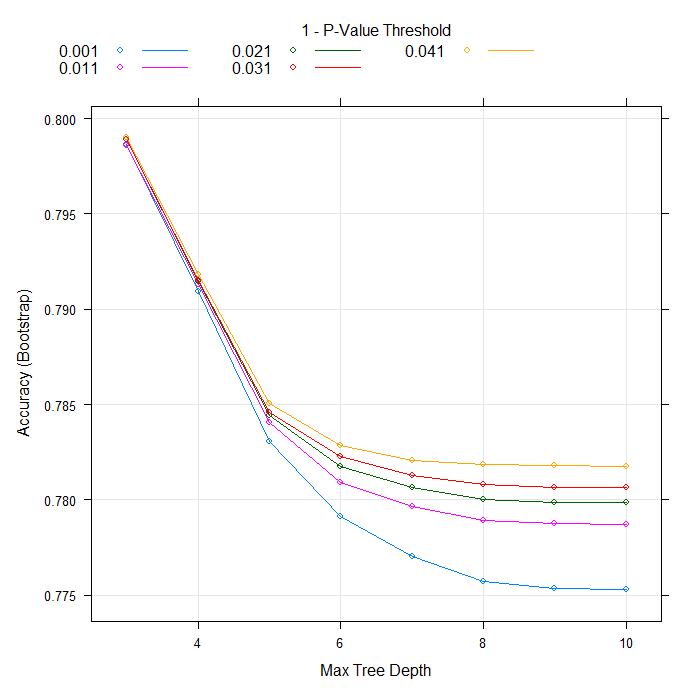

Supplement: Supplementary file 1 [file cancers-14-06116-s001.zip › Supplementary Figure S4.png]
